# Physical Activity and Nutrition Survey and Evaluation for Public Health

**DOI:** 10.3390/nu15194139

**Published:** 2023-09-25

**Authors:** Roberto Pippi, Matteo Vandoni, Carmine Giuseppe Fanelli

**Affiliations:** 1 Centro Universitario Ricerca Interdipartimentale Attività Motoria (C.U.R.I.A.Mo.), Department of Medicine and Surgery, University of Perugia, 06126 Perugia, Italy; carmine.fanelli@unipg.it; 2 Laboratory of Adapted Motor Activity (LAMA), Department of Public Health, Experimental Medicine and Forensic Science, University of Pavia, 27100 Pavia, Italy; matteo.vandoni@unipv.it

Regular physical activity (PA) and healthy nutrition are effective strategies to improve crucial modifiable lifestyle factors that affect health status, both in healthy people and in special populations suffering from metabolic disorders (e.g., obesity and type 2 diabetes, or DM2) or other Non-Communicable Diseases (NCDs) [1]. For this reason, it is mandatory to provide guidance on the proper implementation of a healthy lifestyle by suggesting nutritional medical therapies and prescribing exercise programs to improve people’s health. The evaluation of these modifiable lifestyle factors is also a mandatory component of every medical visit or intervention.

In the Nutrients Special Issue “Physical Activity and Nutrition Survey and Evaluation for Public Health” (https://www.mdpi.com/journal/nutrients/special_issues/physical_activity_nutrition_survey_evaluation (accessed on 22 September 2023)), we presented some papers to focus on the importance of evaluation through surveys, tests, or other forms of evaluation in different settings (i.e., sport and clinical practice) to better orient interventions for public health improvement. This Editorial aims to summarize the twelve articles (nine original articles, one review, and two systematic reviews) that are published in this Special Issue.

Among the behavioral patterns linked to people’s health is the Mediterranean Lifestyle (MLS), which includes a high adherence to the Mediterranean Diet (MD) and regular physical activity. Montero-Sandiego et al. [2] presented a systematic review of different methodologies and tools to assess the MLS, and the authors identified four indexes (MEDiLIFE-index, MEDI-Lifestyle, Total Lifestyle Index, and MedCOVID-19 Score). As a consensus has not yet been reached on a single instrument to comprehensively and reliably measure the MLS that has proven and adequate psychometric properties. The authors emphasized the need to design an instrument for the general population that includes all dimensions of the MLS.

Continuing on the theme of Mediterranean nutritional habits, Béjar [3] presented their results on the difference between weekly and weekend dietary patterns that emerged from the evaluation of Mediterranean diet habits in a group of 361 students (263 women and 98 men) of the University of Seville (Andalusia, Spain). Using the Electronic 12-h Dietary Recall smartphone application and calculating the adherence to the Mediterranean diet index, the researchers observed that the diet quality of the entire sample of Spanish university students was poor in general, especially on the weekends. For this reason, these results could be used to improve public health campaigns to promote healthy eating among Spanish university students.

In another paper published in this Special Issue, Trovato et al. [4] analyzed the relationship between PA, sun exposure, vitamin D, and perceived stress in a sample of the general Italian population. The authors identified an association between sunshine/vitamin D only in those who are physically active, suggesting that PA is necessary to realize the benefits of sunshine/vitamin D supplementation for stress.

Some studies of this Special Issue focused on various PA and nutrition aspects that need to be considered when we talk about PA evaluation and prescription. Regarding PA and exercise prescription for health, Calcaterra et al. [5] emphasized the importance of the modality and volume of exercise required to obtain benefits. The authors assessed clinical, auxological, hemodynamic (i.e., weight, height, waist circumference, waist-to-height ratio, visceral adiposity index, fasting blood glucose, lipid profile), and lifestyle parameters (i.e., PA levels, etc.) in a group of 35 children (14 females/21 males) with obesity participating in 12 weeks of online exercise training. The participants were allocated into two groups based on the volume of performed exercise (above or below 1200 MET·min/week). The authors concluded that improvement in the volume of the structured exercise was associated with a reduction in the arterial pressure percentile, and that progress in metabolic controls was evident. Moreover, the online exercise training program, when designed and progressively adapted according to individual fitness level, was a successful instrument and alternative to promote health improvements because it is sustainable both economically and organizationally.

Another alternative card to play in the clinical field is proposed by Pippi et al. [6] with an article examining an aquatic exercise program in 93 adults (72 women and 21 men) with obesity and/or DM2. The paper utilized the well-established evaluation model developed by the C.U.R.I.A.Mo. Healthy Lifestyle Institute of Perugia University (Umbria, Italy). This study showed the effectiveness of an aquatic exercise program, supervised by experts such as kinesiologists, in improving body mass index, waist circumference, and blood pressure values in obese adults with and without type 2 diabetes. The authors recommended the usefulness of aquatic exercise in managing patients with metabolic diseases who often present with other health impairments, such as musculoskeletal problems or cardiovascular or rheumatic disease that could contraindicate gym-based exercise.

Petri et al. [7] performed a body composition assessment in a group of 68 participants (41 males aged 30.1 ± 9.2 years and 27 females aged 32.1 ± 8.0 years) at the 2021 World Natural Bodybuilding Federation Italian Championships using the vector bioimpedance methodology (BIVA). The study aimed to provide normative references of body composition with the BIVA and to compare BIVA assessments performed on both sides and the upper and lower body. They concluded that BIVA references in bodybuilders could help adjust their training and nutritional programs during the peak week before a competition.

Kasović et al. [8] evaluated the acute effects of a four-week resistance training program on body composition, muscular fitness, and flexibility in 764 Croatian veterans. The participants were subdivided into two groups (50–64 and 65–80 years old). Based on their results which showed that resistance training increased lean mass, muscular fitness, and flexibility and decreased body weight, body mass index, and fat mass across the groups, the authors concluded that even a relatively short resistance training intervention may lead to higher physical fitness levels in men and women aged 50–80 years.

The “lockdown” and social isolation imposed in some world countries during the SARS-CoV-2 virus global health crisis period led to a drastic alteration in lifestyle habits, which was particularly dangerous for people with NCDs. In this Special Issue, two contributions linked to COVID-19 were presented. Ke et al. [9] presented a study that described an online survey result of PA, dietary behavior, and body weight changes during the COVID-19 nationwide lockdown in Taiwan. The results were obtained from the replies of 374 respondents, aged between 20 and 66, and showed a negative impact on all PA levels of participants, with a significant increase in a sedentary lifestyle. However, the authors reported that some good eating habits, including eating fewer snacks or sweets and being less likely to eat as a reward, significantly increased during this period. This may also explain the lack of significant changes in body weight during the lockdown period. Another contribution to determining the training and nutrition plans of professional athletes after infection with COVID-19 was provided by Sliz et al. [10]. The authors described the assessment of the consequences of SARS-CoV-2 virus infection and pandemic restrictions on nutrition and PA among 49 endurance athletes (43 male and 6 female), and its validation through cardiopulmonary exercise testing. This paper underlined the importance for nutritionists considering which eating habits emerge when athletes have limited training opportunities and spend more time at home (i.e., adequate supply of fluids, fruits and vegetables, vitamins, and whole grains), and that trainers should be more aware of body and performance changes after suffering from COVID-19. In addition, another lesson that the COVID-19 era has taught us is the importance of technology. Considering that wearable devices are increasingly popular in the clinical and non-clinical population as a tool for exercise prescription, the monitoring of daily physical activity and nutrition, and health-related parameter management, Natalucci et al. [11] provided a narrative review of the effectiveness of new technologies in physical activity and nutrition. The authors aimed to review the current application of wearable devices in NCDs, highlighting their role in prescribing and monitoring daily PA and dietary habits in a population living with chronic diseases. This study concluded that the benefits obtained from the use of wearable devices are likely to translate into public health and are important tools for the development of prevention plans in everyday life and clinical practice for optimal patient management.

Although proper nutrition and adequate PA are recognized as the most important factors promoting health, the cult of slimness and excessive attention to a healthy lifestyle in the interaction of some factors can sometimes lead to eating disorders. Orthorexia, in particular, is an informally diagnosed condition characterized by an obsessive preoccupation with eating healthily. This condition is frequently associated with high levels of PA to maintain fitness and health in order to achieve the “ideal” body shape that is promoted by mass media. These themes were discussed by Grajek et al. [12] in their paper evaluating the prevalence of orthorexic behaviors in a group of 300 Polish students in terms of their differential health behaviors, such as diet and PA levels. The authors speculated that students who participate intensely in sports may have a significantly higher exposure to orthorexia than those with lower levels of PA, and they confirmed that orthorexia may occur in students with a low BMI index.

Finally, coaches and clinical team professionals caring for the general population or patients with NDC should better understand the various types of barriers people face that prevent them from adhering to a healthy lifestyle. With regard to difficulties for the general population in adhering to PA and healthy nutrition recommendations, Cavallo et al. [13] presented a systematic review to highlight the barriers that patients with chronic-degenerative diseases experience in implementing a healthier diet and an exercise-based therapeutic program. The authors identified barriers for both for exercise and nutrition including lack of time, environmental barriers, health status, and psychological barriers.
